# The intersections of palliative care and homelessness in social policy: A content analysis of Canadian policy documents

**DOI:** 10.1186/s12904-025-01866-4

**Published:** 2025-09-24

**Authors:** Jeffrey J. Walsh, Tamara Sussman, Harvey Bosma, Rachel Z. Carter, Émilie Cormier, Sarah L. Canham

**Affiliations:** 1 School of Social Work, McGill University, Montreal, Canada; 2 Department of Gerontology, Simon Fraser University, Vancouver, Canada; 3 School of Social Work, University of British Columbia, British Columbia, Canada; 4 Palliative Care Programs, Providence Health Care, Vancouver, Canada; 5 Department of Medicine, Faculty of Medicine, University of British Columbia, British Columbia, Canada; 6 Département de psychologie, Université du Québec à Montréal, Montréal, Canada; 7 College of Social Work, University of Utah, Salt Lake City, USA

**Keywords:** Equity, Shelter, End-of-life, Bereavement, Housing, Homelessness, Content analysis

## Abstract

**Background:**

Palliative care for people experiencing homelessness (PEH) is a social issue of increasing importance. Policymakers are best positioned to lead societal responses by naming the issue in policy documents, allocating resources to address palliative care for PEH, and creating frameworks or guiding principles to inform action. This study aims to examine how, if at all, policymakers in Canada are identifying and addressing the issue of palliative care for diverse PEH in policies and frameworks governing the palliative care and/or homelessness sectors.

**Methods:**

We conducted a content analysis of 75 Canadian policy documents governing palliative care or homelessness for the presence of discussion of homelessness (in palliative care documents) and end-of-life (in homelessness documents). The level of discussion (no, indirect, minimal, significant), the jurisdictional level (municipal/city, provincial/territorial, national), and mention of intersecting identities were also recorded.

**Results:**

Of the 75 documents analyzed, 42 contained no discussion of palliative care and homelessness, and only five contained significant discussions by explicitly identifying barriers, describing unique needs, and identifying competencies or innovative practices to promote access and inclusion. All significant or national level discussions were palliative care documents. Intersectional discussions of palliative care for PEH were found in 9 of 75 of documents, with ethnicity and Indigeneity mainly mentioned in palliative care documents, and older age and gender mentioned solely in homelessness documents.

**Conclusions:**

There are critical gaps in Canadian policy documents governing palliative care and homelessness. Most policy documents fail to name or address the issues, with the gap most pronounced in homelessness documents, which contained no national level or significant discussions about end-of-life. Additionally, policy documents from both sectors seldomly discussed the unique needs and barriers of older, racialized, and/or gender-marginalized PEH at end-of-life. While competencies and service level solutions appear to be emerging within palliative care policies at the national level, policymakers from both sectors and across all levels of government must collaborate to address the unique needs of diverse PEH at end-of-life.

**Supplementary Information:**

The online version contains supplementary material available at 10.1186/s12904-025-01866-4.

## Background

Palliative care is an integral component of the healthcare continuum for all people, including people experiencing homelessness (PEH). Palliative care seeks to provide holistic care by addressing the physical, psychological, social, and spiritual needs of individuals with life-limiting conditions and the people who support them [1]. While there is a growing movement to activate a palliative approach to care once a life limiting diagnosis is made, these principals of care are all the more pertinent in the advanced stages of a condition when end-of-life and bereavement supports are required to enhance comfort and ensure quality of care during and following death [1–3].

The provision of palliative care for PEH is a social issue of increasing importance for policymakers, healthcare decision-makers, service providers, and community advocates across the globe. Despite economic prosperity in many OECD (Organisation for Economic Co-operation and Development) countries, the number of PEH continues to grow [4–7]. This growing segment of the population includes both visibly homeless people (i.e. living on the streets or in encampments), as well as a larger number of invisibly homeless people living in temporary, unstable, unsafe, or inadequate spaces such as emergency shelters, substandard buildings, and couch-surfing with loved ones [4, 5]. The impacts of homelessness on the physical and emotional wellbeing of PEH are tremendous as homelessness causes increased rates of chronic illness [8], premature frailty [9], and decreased life expectancy [10, 11], resulting in an increased need for palliative care among this population.

Despite their increased need for palliative care, PEH often face significant barriers in accessing palliative care and experience disparities in palliative outcomes [12–14]. For example, most community-based palliative care programs focus on providing care for people who have stable housing and commonly exclude vulnerably housed people living in encampments, shelters, or buildings deemed inadequate or unsafe [15, 16]. Likewise, numerous personal and social experiences create barriers to care. Mistrust of healthcare providers due to experiences of violence, including racism, classism, and colonialism, result in PEH delaying or avoiding care [17, 18]. Similarly, many palliative care systems are intolerant of substance use and severe mental illness – both of which are social circumstances found in higher prevalences in homeless populations [19] – resulting in fragmented palliative care for this population [15, 20].

While issues of mistrust, substance use, and mental health present barriers for many palliative care programs, homeless services have developed particular expertise in these issues with this population [9, 13, 21]. As such, homeless services often serve as de facto palliative care providers for PEH at end-of-life [9, 13, 21] However, homeless service providers often lack the capacity to provide comprehensive palliative care due to limited training, and a lack of resources to support the increased care needs that emerge at end of life [13, 21]. This leaves PEH without access to palliatively trained staff and resources compared to what stably housed persons can access. With an incongruence between need and access, there is an urgency for decisive action and guidance from palliative and homelessness leaders to integrate a palliative approach to caring for PEH.

One mechanism used to address social issues warranting attention and action is policy. Outlining specific social issues within policy can result in increased visibility and priority setting, greater accountability on the part of organizations, communities, and governments to address these issues, and an augmented allocation of resources [22, 23]. As such, the degree to which the intersection of homelessness and palliative care is included in policies is crucial to ensure that the unique needs of PEH are being prioritized and considered in strategies, frameworks, and action plans shaping service delivery in both sectors.

To reflect the diversity of PEH in need of palliative care an intersectional perspective is also required. Intersectionality provides such a framework by helping to illuminate how differing social identities and circumstances within a social hierarchy create varied experiences of inclusion and exclusion [24]. An intersectional lens applied to palliative care for PEH forces us to view access not as universal, but rather shaped by the social circumstances of age, gender, class, health status, or race and ethnicity. For example, our engagement with a larger Canadian study on aging in place for older homeless adults has enforced the unique needs and experiences of aging while homeless, a phenomena of growing significance globally [4, 5, 25]. Additionally, colonialism and systemic racism are acknowledged as playing an immense role in this area by creating an overrepresentation of migrant, racialized, and Indigenous Peoples in homelessness [4, 6, 7] as well as inequities in access to palliative care [17, 18].

We therefor engaged in our analysis of social policies governing homelessness and palliative care by asking the following three interrelated research questions: (1) How, if at all, is homelessness discussed in Canadian palliative care policies, (2) how, if at all, is palliative care discussed in Canadian homelessness policies, and finally (3) how, if at all, do all documents discussing the intersections of homelessness and palliative care attend to the diverse social identities and circumstances of PEH such as older age, gender, and/or race or ethnicity.

## Methods

We employed a summative content analysis of policy documents to meet our aims [26]. This approach is well suited for analyzing key ideas and messages embedded within large documents, and for describing if and how documents engage with a topic of interest [26–28]. The precise steps we used for identifying and analysing policies are described below.

### Step one: identifying inclusion & exclusion criteria

We searched for Canadian policy documents focused solely on palliative care or homelessness, and adults or older adults. We elected to limit our search to action plans, strategic plans, laws, and other guiding documents used by organizations in a service region to direct programmatic models or operational procedures [29, 30]. These higher-level policies are the most influential for guiding practice and service allocation in a given region [29, 30]. We included documents written in either English or French as these are the two official languages used by government agencies in Canada. Policy documents that focused on children or youth, or on broader health and social concerns, such as aging or general housing strategies, were excluded.

### Step two: compiling & screening relevant policy documents

Policy documents were identified using a search strategy for publicly available Canadian policies developed in consultation with a McGill University librarian. Our strategy used two search engines: Policy Commons and Google. Policy Commons, a large, centralized database of public policies and grey literature from various governmental and community-based organizations, allowed for a broad and comprehensive search. Google was used to conduct geographically focused searches for relevant policy documents and to identify any documents not found through Policy Commons. We conducted an initial search for policy documents in Spring 2022 and repeated our search in Fall 2024 when we finalized our protocol.

Both Policy Commons and Google searches included terms for policy documents, as well as either palliative care or homelessness terms. To find policy documents, we used the search string “policy OR framework OR strategy OR law OR legislation OR standard OR statement OR guideline”. To identify palliative care and homelessness documents, we included the terms “palliative care” OR “end-of-life care” and “homeless*” OR “houseless*” respectively.

Our Google search strategy considered Canada’s unique geo-political context. Canada is a geographically large and diverse country with policies and programs administered largely through three jurisdictions of government: national/federal (e.g. Canada), provincial/territorial (e.g. Ontario), and municipal/city (e.g. Toronto). We used the Google search engine to conduct separate searches using terms for national, all provinces or territories, and major Canadian cities with the largest homeless populations. Conducting separate targeted Google searches for each of these geographic areas addressed a limitation when using the Google search engine, as it only displays the results their algorithm deems most relevant.

In Canada, palliative care falls solely within national and provincial/territorial government jurisdiction, so we conducted searches for palliative care documents using national and provincial/territorial terms. On the other hand, homelessness, while largely funded by the national and provincial/territorial governments, is commonly addressed through policies developed at the municipal/city. Thus, in addition to conducting searches using the national and provincial/territorial terms, we also conducted policy document searches for cities with the highest proportion of people experiencing homelessness, such as Toronto, Vancouver, and Montreal. Since the provinces of Québec and New Brunswick use French as their official language, we expected that some policy documents could be only available in French. A bilingual Francophone co-author translated the English search terms to French and conducted French-based Google searches for documents in these regions.

**Fig. 1:**
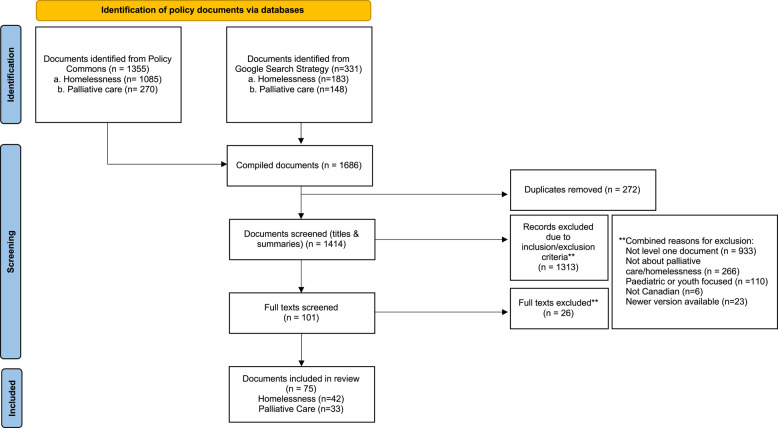
PRISMA flow chart depiciting data base search and screening of policy documents

Our initial search yielded 1686 results (see PRISMA, Fig. 1): 1355 from Policy Commons, 331 from Google. Title and summary reviews resulted in the removal of duplicates (*n* = 272) and policies that did not meet our inclusion criteria (*n* = 1313). Full-text screening resulted in the removal of a further 26 documents that did not meet inclusion criteria. In total, 75 documents were retained for analysis: 33 palliative care and 42 homelessness documents.

### Step three: data extraction

We used keyword searches to identify relevant passages in policy documents for data extraction and further analysis. We identified most keywords prior to data extraction, but as we became familiar with the documents, we developed additional keywords for a more comprehensive analysis of all documents. Initial keywords capturing homelessness included: homeless, hous*, street, and shelter. Homelessness related keywords added during data extraction included: mental, substance, addict*, and equit*. Keywords capturing palliative care included: end-of-life, palliative, hospice, death, dying, morbid*, and terminal. Palliative related keywords added during data extraction included: health, illness, CPR, and resus*. Verbatim excerpts that were identified via keyword searches were extracted and transferred to an Excel Spreadsheet for further analysis. Document characteristics, such as year of publication, geographic location, and jurisdictional level (national/federal, provincial/territorial, or municipal/city) were also recorded.

### Step four: content analysis

We analysed our extracted data in a series of steps. First, based on extracted passages, we coded policy documents by their level of discussion ranging from “no discussion”, “minimal discussion”, or “significant discussion” – a categorization used by one of the authors in another study on Canadian housing policies [31]. Palliative care documents which did not name homelessness, and homelessness documents which did not name palliative care were coded as “no discussion”. Documents that explicitly named palliative care and homelessness without further discussion or elaboration were coded as “minimal discussion”, whereas documents which included description of or elaborated on issues or practices in palliative care for PEH were coded as “significant discussion”.

In the second stage of analysis, we skimmed the policies coded as “no discussion” and discovered some of these documents touched on issues related to palliative care, end-of-life, bereavement and/or homelessness indirectly by including language related to health (in homelessness policies) or equity (in palliative care policies). For example, some palliative care documents mentioned sub-populations experiencing disparities in palliative care access or acknowledged underserved populations. Likewise, some homelessness documents discussed higher rates of chronic illness, premature death, and bereavement following death of a spouse – concerns related to palliative care. We therefore created a fourth level of discussion code called “indirect discussion” and re-coded some documents formerly coded as “no discussion.”

In the third stage, we analyzed the content of extracted passages quantitatively and qualitatively for each discussion level across sectors (palliative care/homelessness) and jurisdiction (national, provincial/territorial, municipal/city). Quantitatively, we recorded the overall frequencies documents mentioned keywords explicitly about palliative care for PEH (use of palliative care keywords in homelessness documents and use of homelessness keywords in palliative care documents). Qualitatively, we analyzed each extracted passage to identify and code the main topics discussed related to palliative care and homelessness, as well as their inclusion of intersecting identities such as ethnicity, older age, or gender, when discussing PEH requiring palliative care.

## Results

In total, 75 policy documents were included: 33 palliative care documents and 42 homelessness documents (see Supplementary Material for document list and Table 1 for summary numbers). Of the 33 palliative care documents, 11 (33.3%) contained no mention of homelessness, nine (27.3%) contained indirect discussion, eight (24.2%) contained minimal discussion, and five (15.2%) contained significant discussion. Of the 42 homelessness documents, 31 (73.8%) contained no mention of palliative care, four (9.5%) contained indirect discussion, and seven (16.7%) contained minimal discussion. No homelessness documents discussed palliative care significantly.

**Table 1:** Document characteristics by level of discussion

	Level of Discussion				Total
	Significant	Minimal	Indirect	None	
**Total** n (%)	5 (6.7)	15 (20)	13 (17.3)	42 (56)	75 (100)
# Mentions - mean (min-max)	7.4 (2–17)	2.6 (1–8)	0 (-)	0 (-)	-
Jurisdictional Level					
National	4	3	2	5	14
Provincial/Territorial	1	6	8	14	29
Municipal/city	0	6	3	23	32
**Palliative Care** n (%)	5 (15.2)	8 (24.2)	9 (27.3)	11 (33.3)	33 (100)
# Mentions - mean (min-max)	7.4 (2–17)	2.86 (1–6)	0 (-)	0 (-)	-
Jurisdictional Level					
National	4	3	1	3	11
Provincial/Territorial	1	5	8	7	21
Municipal/city	0	0	0	1	1
**Homelessness** n (%)	0 (0)	7 (16.7)	4 (9.5)	31 (73.8)	42 (100)
# Mentions - mean (min-max)	0 (-)	2.29 (1–8)	0 (-)	0 (-)	-
Jurisdictional Level					
National	0	0	1	2	3
Provincial/Territorial	0	1	0	7	8
Municipal/city	0	6	3	22	31

For documents with any level of discussion of palliative care and homelessness (*n* = 33), nearly half (*n* = 15) were provincial/territorial jurisdiction. The remaining documents were evenly split between national and municipal/city documents (*n* = 9 each). The jurisdictional level of the documents varied between the palliative care and homelessness documents. Of the 75 policy documents identified, only 14 documents were at the national level. Of these 14 documents, most (*n* = 11) were palliative care documents, most (*n* = 9) included some level of discussion of palliative care and homelessness, and some (*n* = 5) included significant discussion. Of the 32 municipal/city-level documents, almost all were homelessness documents (*n* = 31) and most (*n* = 23) had no discussion of palliative care.

Documents containing indirect, minimal, or significant levels of discussion of palliative care and homelessness discussed a range of topics summarized below and in Table 2.

**Table 2:** Topics by level of discussion

Topic	Level of Discussion
Equitable access to palliative care for all	Indirect - Palliative care
Populations with reduced access to palliative care	Indirect - Palliative care
Increased chronic health and/or premature mortality for PEH	Indirect - Homelessness
Bereavement and risk of homelessness	Indirect - Homelessness Minimal - Homelessness
Unique palliative needs for PEH	Minimal - Palliative care Minimal - Homelessness
Barriers to palliative care for PEH	Minimal - Palliative care Significant - Palliative care
Settings of care for PEH	Minimal - Palliative care Significant - Palliative care
Interventions or practices for PEH	Minimal - Homelessness Significant - Palliative care
Calls to action to improve palliative care for PEH	Significant - Palliative care

### Indirect discussion

In total, 13 documents contained indirect discussion of topics related to homelessness or palliative care among PEH, comprising nine palliative care documents and four homelessness documents.

#### Indirect discussion of homelessness in palliative care documents

Indirect discussions of homelessness in palliative care documents (*n* = 9; 8 provincial/territorial, 1 national) mentioned equity and palliative care access for equity-deserving populations, including people living with mental illness or substance use concerns - both of which are established causes and consequences of housing precarity [32–40]. Most documents (*n* = 8) implied some groups do not have access to palliative care and stated that equitable access to palliative care needs to be considered, but did not specifically mention PEH [32–39]. For example, one provincial law mandated their health department to develop a framework which: “identifies measures to facilitate equitable access to palliative care across Ontario, with a focus on underserved populations” [32]. Barriers to support faced by the bereaved were also implied by mentioning “the need to consider how equitable access to bereavement supports can be established” [35].

Some policy documents named populations commonly associated with homelessness as having unique palliative care needs, but these documents did not directly mention homelessness. For example, two documents mentioned that people experiencing mental health and substance use challenges face unique palliative care needs without further elaboration [33, 40]. These discussions of mental health and substance use were less frequent in these documents than broader discussions of equity.

#### Indirect discussion of palliative care in homelessness documents

Indirect discussion of palliative care in homelessness documents (*n* = 4; 3 municipal/city, 1 national) included mention of at least one of the following related to homelessness: higher rates of chronic illness, premature death, or bereavement [41–44]. Three documents highlighted the elevated threat of death facing PEH [41–43]. One document stated, “[t]he death rate for homeless people is eight to ten times higher than housed people of the same age” and that a lack of housing results in “increased illness and premature death” [41]. Another stated that chronic homelessness increased the “likelihood that [PEH’s] physical and mental health will deteriorate and there is an increased chance of an early death” [42]. Two documents discussed the increased risk of homelessness facing older adults following spousal death. Both documents discussed age by mentioning that older adults were at a higher risk of losing their housing following death of their spouse [43, 44].

### Minimal discussion

Fifteen documents contained minimal discussion of homelessness and palliative care – eight palliative care and seven homelessness documents. These documents spanned municipal/city (*n* = 6, all homelessness), provincial/territorial (*n* = 6; *n* = 5 palliative care, *n* = 1 homelessness), and national (*n* = 3, all palliative care) jurisdictions.

#### Minimal discussion of homelessness in palliative care documents

Mention of homelessness in the eight palliative care documents discussed three topics: unique needs; barriers to accessing palliative care; and settings of palliative care [45–52]. These discussions frequently included PEH only in broad lists with other equity-deserving populations such as Indigenous Peoples or incarcerated people, or settings of care [45–49, 51, 52]. Other than suggesting palliative care providers should consider these populations or settings, the documents did not provide further description or elaboration on the topic.

Three documents identified PEH as having unique palliative care needs without providing further explanation or elaboration of those specific needs [48, 50, 51]. For example, one document listed “street people” [48] as one of several populations with unique palliative needs but did not describe these unique needs. Another document mentioned that palliative care providers were meeting with homelessness service providers to address the unique needs of PEH, but without elaboration on these partnerships, needs, or outcomes [50].

Barriers or disparities in accessing palliative care for PEH were mentioned in three palliative care documents [47, 49, 51]; in some cases, PEH were only listed alongside other equity-deserving populations facing barriers to palliative care. For example, one document acknowledged that competent palliative care nurses “identifie[d] barriers to palliative care for vulnerable or marginalized populations, including, but not limited to: the homeless, [I]ndigenous [P]eoples, those who are incarcerated and those living in rural communities” [47]. Other documents described equity-deserving populations, including PEH, that had reduced access to palliative care. One document stated: “Disparities in access to palliative care are faced most often by those…with lower socioeconomic status, and/or underserved populations, which includes homeless and/or vulnerably housed people*…*” [49]. Homeless populations are identified in these lists as experiencing difficulties when accessing palliative care, but further discussion or strategies are missing.

Four palliative care documents mentioned settings of palliative care and death for PEH, including the streets and homeless shelters [45, 46, 51, 52]. It was highlighted that PEH die in these settings without appropriate palliative care [46]. Multiple documents used identical phrasing to define settings of care. For example, several documents defined it as:

[T]he location where care is provided. Settings of care may include the person’s home, primary care settings (e.g., a doctor’s office, nursing station, community clinic), an acute, chronic, or long-term care facility, a hospice or palliative care unit, a jail or prison or in the case of homeless individuals, the street [45].

In these definitions, palliative care settings were expanded to homeless spaces by including them in these broader lists, but further implications or strategies for providing care in these settings were not included.

#### Minimal discussion of palliative care in homelessness documents

Though minimal, seven documents discussed palliative care in homelessness documents focused on two areas: palliative care needs of PEH and broader homelessness practices or interventions potentially benefiting PEH with palliative care needs [53–59]. Five homelessness documents mention palliative care needs of PEH, but provided no further description of these needs [53, 55, 57–59]. Two documents used identical phrasing to highlight that “older persons who experience homelessness may suffer from chronic illness, loss of mobility, or may be in need of palliative/end-of-life care more frequently than other homeless persons” [53, 57]. One document additionally stated that death of a spouse leads to increased risk of homelessness for surviving spouses, a discussion also found in homelessness documents with indirect discussion of palliative care [53]. Another document framed the “unmet needs at end-of-life” of PEH in the context of colonialism and vulnerable housing, but did not elaborate on these needs [55].

Palliative care was also mentioned within the context of practices or interventions for PEH. Two documents mentioned interventions addressing broader homelessness as potentially beneficial for PEH with palliative care needs without explaining further [54, 56]. First, PEH with palliative care needs were noted as potentially benefiting from integrated wrap-around homelessness case management [56]. Second, “place-based [permanent supportive housing] models may also be operated to provide special care, such as long-term care facilities or palliative programs” [54]. Neither of these documents provided further explanation of how these homelessness services could support PEH at end-of-life.

### Significant discussion

Only five documents (4 national, 1 provincial/territorial) significantly discussed the intersections of homelessness and palliative care, all of which were palliative care documents [1, 60–63]. Discussion of homelessness centred around four topics: settings of care; barriers to accessing palliative care; practices and interventions; and calls to action.

Discussions of settings of care and barriers to accessing palliative care were found in all five documents and did not differ from discussions found in palliative documents coded as minimal level of discussion. These documents listed homeless shelters and the streets as potential sites of palliative care [1, 61, 62] and noted that PEH face barriers accessing traditional palliative care services [1, 60, 61, 63].

Practices and interventions responding to the unique palliative care needs of PEH were discussed in all five documents. These included individual- and system-level responses. Individual-level responses focused on nursing or physician competencies in palliative care related to staff working with PEH, such as skills in advocacy and identifying barriers to palliative care [62]. Other documents included broader competencies, such as the provision of palliative care and capacity building with PEH, within their professional self-assessment tools [63].

System-level responses included the integration of palliative care services into existing homelessness services, and innovative palliative care models [1, 60, 61]. Some documents advocated for the integration of palliative care into homelessness settings, such as shelters and the streets [1, 61]. The current integration of palliative care in existing homelessness spaces was highlighted in one document as “a small number of hospices for the homeless in Canada, units located in shelters…[where] shelter staff receive extensive training and provide round-the-clock care” [61]. These discussions advance the discussions of settings of care found in the minimal discussion palliative care documents, as well as interventions for PEH with palliative care needs found in the minimal discussion homelessness documents.

Two documents discussed innovative programs responding to the unique need of PEH [1, 60]. Palliative care outreach teams and a hospice embedded in a homeless shelter were discussed as reducing palliative care inequities [1]. Emerging community-driven initiatives exploring and responding to the unmet needs of OPEH at end-of-life were discussed as “new resources are being developed to support communities seeking to improve access to palliative care for the homeless and vulnerably housed” [60]. National community-driven initiatives, such as the *Equity in Access to Palliative Care Project*, were identified as exploring and addressing the palliative care needs of OPEH in 20 communities across Canada [60].

Calls to action to improve access to palliative care for PEH were discussed in two documents. One recognized that “there is still work to do to meet the needs of diverse populations and improve access to palliative care,” as well as work to “spread and scale models of care” to address the unique needs of PEH [60]. Another document directly called upon policymakers and palliative care leaders to create policies that integrate best-practices for palliative care into non-traditional settings, such as homeless shelters [61]. This document further questioned the barriers to implementing competent palliative care services in homeless settings across Canada: “If some hospices, some long-term care homes, some home care services, some shelters and some regions can do it, why is this standard of care not available everywhere?” [61]. These calls to action build upon previous discussions of barriers and disparities and directly challenge policymakers and health-systems leaders to innovate practices aimed at increasing equitable access to palliative care for PEH.

### Intersectional analysis

Our intersectional analysis found 17 policy documents mentioning diverse identities in extracted passages discussing palliative care for PEH. Eight documents (all palliative care) only listed diverse identities, such as ethnicity/Indigeneity, gender, older age, and sexuality, alongside PEH without discussing how these identities intersect to shape unique needs, experiences, and barriers to palliative care [1, 47–51, 62, 63]. The remaining nine documents (six homelessness and three palliative care) elaborated on how the intersections of older age, gender, and/or race, ethnicity, or Indigeneity either heighten risk of homelessness or shape palliative care access and experiences. Table 3 provides an overview of documents containing intersecting identities in their discussion of palliative care for PEH.

**Table 3:** Documents including intersecting identities in discussions of palliative care for PEH

Document characteristics			Intersecting Identities		
Name (Year)	Sector	Discussion-Level	Ethnicity or Indigeneity	Gender	Older Age
A Provincial Framework for End-of-life Care (2006)	Palliative Care	Minimal	L	(-)	(-)
The Way Forward National Framework: A Roadmap for an Integrated Palliative Approach to Care (2015)	Palliative Care	Minimal	X	(-)	(-)
The Nova Scotia Palliative Care Competency Framework (2017)	Palliative Care	Minimal	L	(-)	(-)
Pan-Canadian Framework for Palliative and End-of-life Care Research (2017)	Palliative Care	Minimal	X	(-)	(-)
The Framework on Palliative Care in Canada (2018)	Palliative Care	Significant	L	L	L
Ontario Provincial Framework for Palliative Care (2021)	Palliative Care	Minimal	L	(-)	(-)
The Ontario Palliative Care Competency Framework (2019)	Palliative Care	Significant	L	L	L
Blueprint for Action 2020–2025 (2019)	Palliative Care	Minimal	L	(-)	(-)
Palliative care and End-of Life Care: Alberta Provincial Framework Addendum (2021)	Palliative Care	Minimal	L	(-)	(-)
The Framework on Palliative Care in Canada - Five Years Later (2023)	Palliative Care	Significant	X	(-)	L
The Canadian Interdisciplinary Palliative Care Competency Framework (2021)	Palliative Care	Significant	L	L	L
Best Practice Guidelines for Ending Women’s and Girl’s Homelessness (2015)	Homeless	Indirect	(-)	X	X
Red Deer Community Housing and Homelessness 5 Year Integrated Plan (2019)	Homeless	Minimal	(-)	X	X
Everyone is Home: A Five-Year Plan to End Chronic and Episodic Homelessness in Regina (2018)	Homeless	Minimal	(-)	(-)	X
Framework for the Blueprint to End Homelessness in Toronto (2011)	Homeless	Minimal	(-)	(-)	X
Community Plan to End Homelessness in the Capital Region (2019)	Homeless	Minimal	X	(-)	(-)
Kelowna’s Journey Home Strategy (2018)	Homeless	Indirect	(-)	(-)	X

L = List only

#### Older age (*n* = 5)

The most common intersecting identity included in discussions of palliative care for PEH was older age. Five documents, all of which were from the homelessness sector, discussed older age when mentioning palliative care for PEH [43, 44, 53, 57, 58]. Three documents reported that older PEH were at an increased need of palliative care than other age groups experiencing homelessness [53, 57, 58] and three documents suggested that older adults who lose spouses have a heightened risk of homelessness [43, 44, 53] as exemplified in one document stating “[the] risk of homelessness for seniors can also be increased by the death of a spouse” [38]. While these documents imply that older people may simultaneously experience homelessness and bereavement, the call for bereavement supports as a consequence of this risk is notably absent.

#### Gender (*n* = 2)

Two documents discussing older age also contained the only discussions of gender by mentioning that older women face an evaluated risk of homelessness when their spouse dies [44, 53]. One of these documents discussed the interlocking nature of class, age, and gender by highlighting that older bereaved women disproportionately experience an elevated risk of homelessness due to the role spouses played in their financial stability [44]. As with the documents on age, neither document went further to suggest the importance of offering bereavement supports alongside housing.

#### Race, ethnicity, and Indigeneity (*n* = 4)

Four documents discussed race, ethnicity, or Indigeneity intersecting palliative care for PEH (three palliative care and one homelessness). Indigenous Peoples were specifically mentioned in three documents [45, 55, 60], while race and ethnicity were broadly discussed in one document [46]. Discussions focused on two topics: colonialization and systemic racism (*n* = 3) and inclusion of Indigenous community advisors in service planning (*n* = 1). Three documents (two palliative care and one homelessness) discussed colonization and systemic racism as producing palliative inequities for PEH [45, 46, 55]. For example, one homelessness document framed the increased need for palliative care for PEH as a result of colonization of Indigenous peoples [55], while one palliative care document noted that those marginalized due to race or ethnicity more often die in shelters or the streets without palliative care [46]. Lastly, one palliative care document mentioned a community initiative aimed at improving palliative care for PEH as including Indigenous community advisors [60].

## Discussion

As housing insecurity grows and populations age, the palliative care needs of PEH are increasingly important to address in policy and service delivery. While homelessness is an established barrier to most palliative care services [13, 14, 16, 18], our policy document analysis found limited discussion of overcoming these barriers to palliative care for PEH in Canada. While indirect discussions of the broader concepts of equity, disparities, chronic illness, and premature death were more frequent, direct discussions of palliative care and homelessness were found in only a quarter of documents. Additionally, policy documents rarely elaborated on the issues and responses to palliative care for PEH, despite the awareness that this population faces increased rates of chronic illness [8], cancer [64], and early-onset frailty [9]. Policymakers must consider and significantly discuss the palliative care needs of PEH beyond mentioning them in passing or including them in lists alongside other equity-deserving populations. Our analysis found most policy documents lacked an in-depth discussion providing guidance of the issue, and instead only offered broad or vague commentary. Without policy guidance and direction, palliative care and homelessness services are unsupported to address the needs of PEH. Cross-sectoral leadership from policymakers is needed to support those in these sectors.

### Intersectional identities

Older age, gender, and ethnicity and Indigeneity are established social identities intersecting to shape homelessness and palliative care [65]; however, our analysis found few policy documents that meaningfully included them in their discussions. Our analysis further revealed differences between sectors in the social identities included. While historical and ongoing colonization and systemic racism create higher rates of housing insecurity and poorer health outcomes for Indigenous Peoples in Canada [66, 67] and racialized peoples globally [4, 6, 7, 68], inclusion of ethnicity in palliative care discussions for PEH was largely limited to a few palliative care documents. Likewise, older age [4, 5, 25] and gender [44, 69] both produce unique needs and vulnerabilities at end-of-life for PEH; however, only homeless documents mentioned age and gender. Even within these documents, their focus was predominantly on how spousal loss heightens the risk of homelessness without emphasizing the need for bereavement supports alongside housing – an element of support included in palliative approaches to care. Policymakers must address palliative care for PEH intersectionally by discussing the differing needs and vulnerabilities experienced by diverse homeless people further marginalized by racism, sexism, and ageism at end-of-life, and directing necessary resources to these populations.

### Differences in policy documents between sectors

The gap in discussing palliative care and homelessness was more pronounced in documents from the homelessness sector. Our analysis found no homelessness policy documents that significantly discussed palliative care for PEH. Furthermore, the homelessness sector lacks national guidance on palliative care as our analysis found no national homelessness documents with any level of discussion of palliative care. Despite this lack of guidance in homelessness policy, homelessness services play a substantial role in addressing both the medical and psychosocial palliative care needs of PEH [15, 16, 18, 21] without the of training and resources provided in formal palliative care services [13, 21]. Additionally, given the heavy reliance on informal caregivers to care for dying loved ones [14], shelter and street outreach workers often take on the additional work of caring for PEH who are disconnected from their families of origin [13, 69]. National homelessness policymakers need to consider, guide, and allocate resources for the unique palliative care challenges facing PEH with homeless service providers in mind. Researchers can further support this crucial work by exploring the unique palliative care needs of the population, the challenges in addressing these unique needs, and innovative models successfully providing palliative care for PEH. This research could then be utilized by both homeless and palliative care service organizations to develop training modules, care systems, and advocacy strategies.

### Bereavement for PEH in homelessness documents

Homelessness policy documents included in this study mentioned a topic largely absent from palliative care discussions of homelessness – the increased risk of entering homelessness during bereavement especially for older adults and women [69]. The bereavement needs of people new to homelessness are important to acknowledge and address in policy to better support and prevent homelessness for older adults following the death of their spouse.

The unique bereavement of PEH who experience losses while homeless, which were strikingly absent from policy documents, are also critical given the relationship between prolonged homelessness and death [69, 70]. Many PEH who experience the loss of a partner who is also homeless do not meet conventional definitions for common law status, such as cohabitation, due to lack of shelter or restrictive housing policies in single-room occupancies or boarding houses [14]. Rights to property or post-death decision-making may therefore be unacknowledged, particularly if estranged family of the deceased intervene [14, 69]. Legislators and policymakers must create laws and policies which recognize the relationships of PEH despite barriers to recognized cohabitation, and uphold the property, financial, and decision-making rights of PEH following loss of a partner.

### Synergies in policy documents between sectors

Lastly, our analysis found that while discussions in the documents from both sectors rarely overlapped, there were some synergies, including discussion of palliative care needs for PEH and potential interventions. While interventions were discussed in greater detail in palliative care documents, a small number of homelessness documents highlighted homelessness interventions which may additionally support PEH with palliative care needs. Despite both sectors discussing potential interventions, the current discussion of palliative care and homelessness interventions remain disconnected. Palliative care documents focused on the integration of palliative care into existing homelessness services and spaces, such as homeless shelters and street-level services. Conversely, homelessness documents discussed programs addressing homelessness which may unintentionally support PEH needing palliative care, such as supportive housing or integrated case management for people experiencing homelessness. This disconnection creates potential confusion between the sectors, as well as inefficiencies in developing and allocating limited resources.

This incongruence highlights the need for collaborative, cross-sectoral work to create a cohesive national strategy focused on palliative care and homelessness [71]. While these discussions are disjointed, they are a promising initial opportunity for this cross-sectoral work which is already underway between palliative care and homeless service providers in some communities [60]. These findings support the need for a national guiding framework led by the federal government and including diverse community partners to support the alignment and strengthen these cross-sectoral collaborations.

### Limitations

This policy document analysis represents the first analysis of Canadian policy documents regarding the inclusion of homelessness and palliative care in their scope. However, there are several limitations to our study including both the methods used to identify and collect policy documents, as well as the content analysis of the policy documents itself. First, this study only included documents focused solely on homelessness or palliative care; however, as evident with our inclusion of documents coded indirect discussion, these two fields exist within broader social and health contexts. Limiting our analysis to documents focused solely on these issues may have excluded documents that discuss their intersection as a subsection within broader strategies or frameworks. This narrow focus may have also been biased against provinces with smaller homeless populations or more rural populations, which have opted to integrate this discussion within broader health or community frameworks. Future analyses could explore the discussions of palliative care and homelessness, if any, in these documents.

A second limitation relates to our inclusion of only policy documents focused on higher-level governance. This search excluded documents that described service-level operational policies, which may have included important discussions on procedures and interventions in both palliative care and homelessness programs. A third limitation is publication bias as we only included policy documents accessible to the public. This method of identifying and collecting policy documents excludes internal documents and other policy documents not readily available to the public. These documents may include discussions pertaining to palliative care and homelessness. Future research could include methods to identify potential agencies with such policy documents and contact them to try to retrieve such documents.

The fourth and fifth limitations relate to the content analysis itself. The fourth limitation is that we only explored the narrative content of the included policy documents. While policy is integral to driving programs and practices, this analysis did not assess the implementation of policy and how inclusion of these discussions have shaped front-line service provision. Future research could include documents that describe operational policies and procedures and explore the palliative care outcomes, interventions, or resource allocations of governance policies through staff and patient interviews and observations, as well as quantitative research of palliative outcomes.

The final limitation is time – as with any analysis of policy documents, this analysis presents a snapshot in time. It does not include policy documents published after our search for policy documents in Fall 2024 so there may be document missing. Likewise, some policy documents were older – with the oldest document published nearly 20 years ago. Older documents may not represent more recent political changes and realities of their jurisdictions. Conducting a similar policy document analysis in the future, and including the aforementioned staff and patient interviews, particularly with healthcare or government leaders would address these concerns – albeit temporarily.

## Conclusion

This document analysis highlights the limited discussion of homelessness and palliative care in Canadian policy documents. Few policy documents acknowledge the intersection of homelessness and palliative care, with even fewer documents elaborating on issues and interventions to support the palliative care needs of PEH, particularly at the national level. Furthermore, the documents often omit the diverse needs and experiences of older, racialized, and gender-marginalized PEH. These absences are critical issues given that homelessness services provide substantial support for diverse PEH at end-of-life but with a significant gap in guidance from national policymakers. The disconnects between homelessness and palliative care policy documents were also evident. Some homelessness policies acknowledged the elevated risk of becoming homeless facing many bereaved older adults following the death of a spouse – a conversation absent from the palliative care documents. Conversely, some palliative care documents discussed specific calls to action for policymakers to improve palliative care for PEH – a discussion missing in the homelessness documents. Despite the differences in discussions, documents from both sectors discussed some similar topics – such as interventions. While each sector suggests different interventions, the fact that they are discussing interventions responding to palliative care for PEH represents an area ready for greater collaboration between the two sectors. Ultimately, our findings demonstrate the immense need for greater collaboration and guidance from Canadian policymakers at all levels of government and on both sides of this intersecting issue.

## Supplementary Information

Supplementary Material 1.

## Data Availability

Data is provided within the manuscript or supplementary information files.

## Abbreviations

CHPCA: Canadian Hospice Palliative Care Association
OECD: Organisation for Economic Co-operation and Development
OPEH: Older people experiencing homelessness
USDHUD: United States Department of Housing and Urban Development
