# Supplementary material for: The intersections of palliative care and homelessness in social policy: A content analysis of Canadian policy documents
Source: BMC Palliat Care. 2025 Sep 24;24:229. doi: 10.1186/s12904-025-01866-4 (PMC12462172; doi:10.1186/s12904-025-01866-4)
Supplement: Supplementary file 1 — Supplementary Material 1. [file 12904_2025_1866_MOESM1_ESM.docx]

**Supplementary Tables of Included Palliative Care & Homelessness Documents**

**Supplementary Table 1. Palliative Care Policy Documents**

| **Title**  **(Link)** | **Year** | **Geographic Level (Jurisdiction)** | **Discussion Level (n*)** |
| --- | --- | --- | --- |
| Policy Brief on Hospice Palliative Care: Quality End-Of Life Care? It Depends on Where You Live… And Where You Die (<https://policycommons.net/artifacts/1218017/policy-brief-on-hospice-palliative-care/1771108/>) | 2010 | National | Significant (5) |
| The Framework on Palliative Care in Canada  (<https://www.canada.ca/content/dam/hc-sc/documents/services/health-care-system/reports-publications/palliative-care/framework-palliative-care-canada/framework-palliative-care-canada.pdf>) | 2018 | National | Significant (17) |
| The Interdisciplinary Palliative Care Competency Framework  (<https://s22457.pcdn.co/wp-content/uploads/2021/07/palliative-care-competency-framework-EN.pdf>) | 2021 | National | Significant (2) |
| The Framework on Palliative Care in Canada - Five Years Later  (<https://www.canada.ca/content/dam/hc-sc/documents/services/publications/health-system-services/framework-palliative-care-five-years-later/final-pdf-english-report-to-parliament-palliative-care.pdf>) | 2023 | National | Significant (8) |
| The Ontario Palliative Care Competency Framework: A reference guide for health professionals and volunteers (<https://serpcn.ca/Uploads/ContentDocuments/OPCNCompetencyFramework.pdf>) | 2019 | Provincial (ON) | Significant (5) |
| The Way Forward National Framework: A roadmap for an integrated palliative approach to care  (<https://www.chpca.ca/wp-content/uploads/2024/04/TWF-framework-doc-Eng-2015-final-April1.pdf>) | 2015 | National | Minimal (6) |
| Blueprint for Action 2020 - 2025  (<https://www.virtualhospice.ca/Assets/QELCCC-Blueprint-for-Action-2020-2025-H_20200318094129.pdf>) | 2019 | National | Minimal (3) |
| Pan-Canadian Framework for Palliative and End-of-life care research (<https://www.virtualhospice.ca/Assets/PEOLC_Report_EN(1)_20170605131142.pdf>) | 2017 | National | Minimal (1) |
| A Provincial Framework for End-of-life Care  (<https://www.health.gov.bc.ca/library/publications/year/2006/framework.pdf>) | 2006 | Provincial (BC) | Minimal (1) |
| Creating An Integrated Hospice Palliative Care System In Ontario (<https://hpco.ca/qhpcco/Creating_an_Integrated_HPC_System_in%20Ontario_-_29_Nov_2010.pdf>) | 2010 | Provincial (ON) | Minimal (1) |
| The Nova Scotia Palliative Care Competency Framework: A Reference Guide for Health Professionals and Volunteers (<https://library.nshealth.ca/ld.php?content_id=34202519>) | 2017 | Provincial (NS) | Minimal (4) |
| Ontario Provincial Framework for Palliative Care  (<https://files.ontario.ca/moh-ontario-provincial-framework-for-palliative-care-en-2021-12-07.pdf>) | 2021 | Provincial (ON) | Minimal (4) |
| **Title**  **(Link)** | **Year** | **Geographic Level (Jurisdiction)** | **Discussion Level (n*)** |
| Palliative and End-of-life Care: Alberta Provincial Framework Addendum 2021 (<https://www.albertahealthservices.ca/assets/info/peolc/if-peolc-prov-framework-addendum.pdf>) | 2021 | Provincial (AB) | Minimal (3) |
| A Model to Guide Hospice Palliative Care: Modèle de Soins Palliatifs: Fondè sur les Normes de Pratique et Principes Nationaux  (<https://www.chpca.ca/wp-content/uploads/2024/07/norms-of-practice-eng-web.pdf>) | 2013 | National | Implicit |
| The Provincial End-of-life Care Action Plan for British Columbia: Priorities and Actions for Health System and Service Redesign (<https://www.health.gov.bc.ca/library/publications/year/2013/end-of-life-care-action-plan.pdf>) | 2013 | Provincial (BC) | Implicit |
| Palliative and End-of-life care: Alberta Provincial Framework 2014  (<https://www.albertahealthservices.ca/assets/info/seniors/if-sen-provincial-palliative-end-of-life-care-framework.pdf>) | 2014 | Provincial (AB) | Implicit |
| Integrated Palliative Care: Planning for Action in Nova Scotia  (<https://novascotia.ca/dhw/palliativecare/documents/integrated-palliative-care-strategy.pdf>) | 2014 | Provincial (NS) | Implicit |
| An Act Providing for the Development of a Provincial Framework on Palliative Care (bill 3) (<https://www.ola.org/sites/default/files/node-files/bill/document/pdf/2020/2020-12/b003ra_e.pdf>) | 2020 | Provincial (ON) | Implicit |
| Palliative Care: Position Statement  (<https://www.bcnu.org/AboutBcnu/Documents/position-statement-palliative-care.pdf>) | 2021 | Provincial (BC) | Implicit |
| Plan d'action 20-25 Pour un Accès Équitable à des Soins Pallatifs et de Vie de Qualité (<https://publications.msss.gouv.qc.ca/msss/document-003444/>) | 2022 | Provincial (QC) | Implicit |
| Loi Concernnant les Soins de Fin de Vie  (<https://www.legisquebec.gouv.qc.ca/fr/document/lc/s-32.0001>) | 2024 | Provincial (QC) | Implicit |
| Palliative Approach to Care: Service Delivery Model for the NWT (<https://www.hss.gov.nt.ca/sites/hss/files/resources/palliative-approach-care-service-delivery-model-nwt.pdf>) | 2018 | Territorial (NWT) | Implicit |
| State of Principles on Physician Assisted Dying  (<https://www.casw-acts.ca/files/attachements/statement_of_principles_on_assisted_death_0.pdf>) | 2016 | National | None |
| Framework On Palliative Care in Canada Act  (<https://laws-lois.justice.gc.ca/eng/acts/F-31.5/page-1.html>) | 2017 | National | None |
| CPHCA Position Statement on MAiD  (<https://www.chpca.ca/wp-content/uploads/2024/07/CHPCAs-Position-Statement-on-MAiD-%E2%80%93-2023-Update.pdf>) | 2023 | National | None |
| End-of-life Framework: Recommendations for a Provincial EOL care Strategy  (<http://www.bcrenal.ca/resource-gallery/Documents/EOL-Framework.pdf>) | 2009 | Provincial (BC) | None |
| Politique Relative aux Soins de Fin de Vie CIUSSS MCQ  (<https://www.ciusssmcq.ca/telechargement/286/politique-relative-aux-soins-de-fin-de-vie/>) | 2015 | Municipal (QC) | None |
| **Title**  **(Link)** | **Year** | **Geographic Level (Jurisdiction)** | **Discussion Level (n*)** |
| Palliative & End-of-life care: An Introduction to Alberta’s Framework (<https://www.albertahealthservices.ca/assets/info/seniors/if-sen-provincial-palliative-framework-public-document.pdf>) | 2016 | Provincial (AB) | None |
| Palliative care in New Brunswick: A person-centred care and Integrated Services Framework (<https://www2.gnb.ca/content/dam/gnb/Departments/h-s/pdf/en/Publications/HealthCare/PalliativeCareNBFramework.pdf>) | 2018 | Provincial (NB) | None |
| BC Centre for Palliative Care: Inter-professional Palliative Competency Framework  (<https://bc-cpc.ca/wp-content/uploads/2019/09/Competency_Framework_May2019.pdf>) | 2019 | Provincial (BC) | None |
| A Framework for Palliative Care Education and Training in British Columbia  (<https://bc-cpc.ca/wp-content/uploads/2020/01/BC-CPC_Education_Training_Framework_Jan2020_WEB-1.pdf>) | 2020 | Provincial (BC) | None |
| Politique en Soins Palliatifs de Fin de Vie  (<https://publications.msss.gouv.qc.ca/msss/fichiers/2004/04-828-02.pdf>) | 2012 | Provincial (QC) | None |
| Yukon Palliative Care Framework  (<https://yukon.ca/sites/yukon.ca/files/hss/hss-imgs/palliativecareframework.pdf>) | 2015 | Territorial (YT) | None |

* n is the frequency of keywords in minimal and significant discussions

**Jurisdiction abbreviation:**

AB = Alberta

BC = British Columbia

MB = Manitoba

NB = New Brunswick

NL = Newfoundland and Labrador

NS = Nova Scotia

NU = Nunavut Territory

NWT = Northwest Territories

ON = Ontario

PEI = Prince Edward Island

QC = Quebec

SK = Saskatchewan

YT = Yukon Territory

**Supplementary Table 2. Homelessness Policy Documents**

| **Title**  **(Link)** | **Year** | **Geographic Level (Jurisdiction)** | **Discussion Level (n*)** |
| --- | --- | --- | --- |
| Framework for The Blueprint to End Homelessness in Toronto  (<https://www.wellesleyinstitute.com/wp-content/uploads/2011/11/Blueprint_TheFrameworkfinal.pdf>) | 2011 | Municipal (Toronto) | Minimal (8) |
| System Planning Framework  (<https://www.calgaryhomeless.com/wp-content/uploads/2021/02/Adult-System-Planning-Framework.pdf>) | 2017 | Municipal (Calgary) | Minimal (1) |
| Everyone is Home: A Five-Year Plan to End Chronic and Episodic Homelessness in Regina (<https://www.regina.ca/export/sites/Regina.ca/home-property/.galleries/PDFs/P2EH.pdf>) | 2018 | Municipal (Regina) | Minimal (1) |
| Red Deer Community Housing and Homelessness: 5 Year Integrated Plan - Technical Report (<https://www.reddeer.ca/media/reddeerca/about-red-deer/social-well-being-and-community-initiatives/housing-and-homelessness/CHHIPTechnicalReport.pdf>) | 2019 | Municipal (Red Deer) | Minimal (2) |
| Community Plan to End Homelessness in the Capital Region 2019-2024  (<https://victoriahomelessness.ca/wp-content/uploads/2020/02/Greater-Victoria-Coalition-to-End-Homelessness-GVCEH-Community-Plan-to-End-Homelessness-2019-2024.pdf>) | 2019 | Municipal (Victoria) | Minimal (2) |
| 10-Year Housing and Homelessness Plan 2020 - 2030  (<https://documents.ottawa.ca/sites/default/files/housingplan20202030.pdf>) | 2020 | Municipal (Ottawa) | Minimal (1) |
| Belonging In BC: A Collaborative Plan to Prevent and Reduce Homelessness - Initial Phase 2022 - 2025 (<https://news.gov.bc.ca/files/BelongingStrategy.pdf>) | 2022 | Provincial (BC) | Minimal (1) |
| The Blueprint to End Homelessness in Toronto  (<https://www.wellesleyinstitute.com/wp-content/uploads/2011/11/TheBlueprintfinal.pdf>) | 2011 | Municipal (Toronto) | Implicit |
| Safe at Home - A Community-Based Action Plan to End and Prevent Homelessness  (<https://yukon.ca/sites/default/files/yhc/yhc-safe-at-home-end-homelessness-report.pdf>) | 2017 | Municipal (Whitehorse) | Implicit |
| Kelowna's Journey Home Strategy: Technical Report  (<https://www.kelowna.ca/sites/files/1/docs/journey_home_technical_report.pdf>) | 2018 | Municipal (Kelowna) | Implicit |
| Best Practice Guideline for Ending Women's and Girl's Homelessness  (<https://londonhomeless.ca/wp-content/uploads/2012/12/Best-Practice-Guideline-for-Ending-Womens-and-Girls-Homelessness.pdf>) | 2015 | National | Implicit |
| Vancouver's Housing and Homelessness Strategy 2012 - 2021: A Home for Everyone  (<https://vancouver.ca/files/cov/housing-and-homeless-strategy-2012-2021pdf.pdf>) | 2011 | Municipal (Vancouver) | None |
| A Place to Call Home: Nipissing District 10 Year Housing and Homelessness Plan 2014-2024 (<https://homelesshub.ca/sites/default/files/A%20Place%20to%20Call%20Home%20(FINAL).pdf>) | 2013 | Municipal (Nipissing) | None |
| **Title**  **(Link)** | **Year** | **Geographic Level (Jurisdiction)** | **Discussion Level (n*)** |
| Homeless Prevention & Housing Plan 2010 - 2024  (<https://pub-london.escribemeetings.com/filestream.ashx?DocumentId=11110>) | 2013 | Municipal (London) | None |
| Saskatoon's Homelessness Action Plan (From Vision to Action)  (<https://shipyxe.ca/wp-content/uploads/2024/07/HomelessnessActionPlan-FinalPrintVersion-2016.pdf>) | 2016 | Municipal (Saskatoon) | None |
| End Homelessness St. John's - Ending Homelessness in St. John's  (<https://homelesshub.ca/sites/default/files/YYT-Community-Plan-2014-2019.pdf>) | 2016 | Municipal (St. John's) | None |
| St. John’s Homeless-Serving System Coordination Framework (<https://homelesshub.ca/sites/default/files/attachments/StJohnsHomelessServingSystem-Final.pdf>) | 2016 | Municipal (St. John's) | None |
| Framework for the GNWT Response to Homelessness Yellowknife: Government of the Northwest Territories (<https://www.yellowknife.ca/en/city-government/resources/City_Council_and_Mayor/Mayor/YK-Homelessness-Road-Map-Action-Plan-FINAL-3.pdf>) | 2016 | Municipal (Yellowknife) | None |
| Nanaimo's Action Plan to End Homelessness 2018 - 2023  (<https://www.nanaimo.ca/docs/public-safety-department/community-social-service-programs/2018-2023-nanaimo-action-plan-to-end-homelessness-dec-1.pdf>) | 2018 | Municipal (Nanaimo) | None |
| Coming Together to End Homelessness – Hamilton’s Systems Planning Framework (<https://www.homelesshub.ca/sites/default/files/attachments/coming-together-to-end-homelessness-report-final-07252019%20%281%29.pdf>) | 2019 | Municipal (Hamilton) | None |
| City of Richmond Homelessness Strategy 2019 - 2029 (<https://www.richmond.ca/__shared/assets/richmond_homelessness_strategy54624.pdf>) | 2019 | Municipal (Richmond) | None |
| Home, Together: Window Essex Housing and Homelessness Master Plan  (<https://www.citywindsor.ca/Documents/residents/housing/housing-with-supports-and-homelessness-prevention/homelessness/FINAL%20Windsor-%20HH%20Plan%20-%20October%203%20'19.pdf>) | 2019 | Municipal (Windsor) | None |
| Connecting the Circle: A Gender-Based Strategy to End Homelessness in Winnipeg  (<https://wcwrc.ca/wp-content/uploads/2019/09/Connecting-the-Circle-Full-Web.pdf>) | 2019 | Municipal (Winnipeg) | None |
| Localized Approaches to Ending Homelessness: Indigenizing Housing First (<https://winnspace.uwinnipeg.ca/bitstream/handle/10680/1727/2019_IUS--Localized_Approaches_Ending_Homelessness_ENG_Final.pdf?sequence=1&isAllowed=y>) | 2019 | Municipal (Winnipeg) | None |
| Kíkinanaw Óma Strategy to Support Unsheltered Winnipeggers  (<https://endhomelessnesswinnipeg.ca/wp-content/uploads/20200630-Kikinanaw-Oma-Strategy-to-Support-Unsheltered-Winnipeggers.pdf>) | 2020 | Municipal (Winnipeg) | None |
| **Title**  **(Link)** | **Year** | **Geographic Level (Jurisdiction)** | **Discussion Level (n*)** |
| Guiding the Fight Against Homelessness: Focus on 2025  (<https://www.calgaryhomeless.com/wp-content/uploads/2021/09/Guiding-the-Fight-Against-Homelessness-Focus-to-2025_condensed.pdf>) | 2021 | Municipal (Calgary) | None |
| Homeless Strategy to Grande Prairie: 2021 - 2023  (<https://cityofgp.com/sites/default/files/2022-01/homelessness_strategy_2021-2023.pdf>) | 2021 | Municipal (Grande Prairie) | None |
| Plan d'action 2024-2026 - Vision en Matière d'itinérance  (<https://www.ville.quebec.qc.ca/apropos/planification-orientations/itinerance/docs/VisionItinerance_brochure_F2.pdf>) | 2023 | Municipal (Ville de Québec) | None |
| Homelessness Services Capital Infrastructure Strategy  (<https://www.toronto.ca/wp-content/uploads/2023/12/8c9e-HSCISinside231011spreadAODA.pdf>) | 2023 | Municipal (Toronto) | None |
| Integrated Homelessness System Action Plan  (<https://www.durham.ca/en/living-here/resources/IEHSD/Durham-Region-Final-Integrated-Homelessness-System-Action-Plan-3.0-with-Apendix.pdf>) | 2023 | Municipal (Durham) | None |
| Homelessness and Housing Services Plan - July 2024  (<https://www.edmonton.ca/sites/default/files/public-files/assets/PDF/Homelessness-Housing-Services-Plan.pdf>) | 2024 | Municipal (Edmonton) | None |
| A Pathway to Home: Surrey Homelessness Prevention + Response Plan (<https://www.surrey.ca/sites/default/files/media/documents/Surrey-Homelessness-Prevention-Response-Plan.pdf>) | 2024 | Municipal (Surrey) | None |
| Navigating Complexity Together: A Roadmap to Functional Zero by 2030  (<https://www.regionofwaterloo.ca/en/living-here/resources/Housing-Services/PECH/Appendix-A---Final-PECH-Report.2024.pdf>) | 2024 | Municipal (Waterloo) | None |
| Bridging the Transition from Homeless to Housed: A Social Justice Framework to Guide the Practice of Occupational Therapists (<https://www.osot.on.ca/docs/practice_resources/Bridging_the_Transition_from_Homeless_to_Housed.pdf>) | 2020 | National | None |
| Reaching Home: Canada's Homelessness Strategy Directives  (<https://housing-infrastructure.canada.ca/homelessness-sans-abri/directives-eng.html>) | 2023 | National | None |
| Policy Recommendations: A Housing and Homelessness Framework for Newfoundland and Labrador (<https://www.homelesshub.ca/sites/default/files/NLHHN%20Housing%20&%20Homelessness%20Framework%20Oct%202010.pdf>) | 2010 | Provincial (NL) | None |
| Politique Nationale de Lutte à l'itinérance  (<https://publications.msss.gouv.qc.ca/msss/fichiers/2013/13-846-03F.pdf>) | 2014 | Provincial (QC) | None |
| Plan d'action Interministériel en Itinérance 2021-2026  (<https://publications.msss.gouv.qc.ca/msss/fichiers/2021/21-846-01W.pdf>) | 2021 | Provincial (QC) | None |
| Action Plan on Homelessness  (<https://open.alberta.ca/dataset/5601187b-7dfc-45ab-8238-8f4c0be3c634/resource/0e21de0d-1248-4594-8268-0d96860723a0/download/css-action-plan-on-homelessness.pdf>) | 2022 | Provincial (AB) | None |
| **Title**  **(Link)** | **Year** | **Geographic Level (Jurisdiction)** | **Discussion Level (n*)** |
| Solutions for Housing and Homelessness Plan - Progress Update  (<https://beta.novascotia.ca/sites/default/files/documents/1-3165/solutions-housing-and-homelessness-plan-progress-update-october-2022-en.pdf>) | 2022 | Provincial (NS) | None |
| ᐊᖏᕋᖃᖏᑦᑐᓕᕆᓂᖅ Angiraqangittuliriniq: A Framework for Action for Nunavut’s Absolute Homeless, 2015 - 2016  (<https://assembly.nu.ca/sites/default/files/TD%20157-4(3)%20EN%20Angiraqangittuliriniq%202015-2016,%20A%20Framework%20for%20Action%20for%20Nunavut's%20Absolute%20Homeless.pdf>) | 2016 | Territorial (NU) | None |
| A Way Home: A Comprehensive Strategy to Address Homelessness in The Northwest Territories  (<https://www.eia.gov.nt.ca/sites/eia/files/a_way_home_-_gnwt_draft_homelessness_strategy.pdf>) | 2023 | Territorial (NWT) | None |

* n is the frequency of keywords in minimal and significant discussions

**Jurisdiction abbreviation:**

AB = Alberta

BC = British Columbia

MB = Manitoba

NB = New Brunswick

NL = Newfoundland and Labrador

NS = Nova Scotia

NU = Nunavut Territory

NWT = Northwest Territories

ON = Ontario

PEI = Prince Edward Island

QC = Quebec

SK = Saskatchewan

YT = Yukon Territory
